# Simultaneous Quantitative Determination of Synthetic Cathinone Enantiomers in Urine and Plasma Using GC-NCI-MS

**DOI:** 10.1155/2018/4396043

**Published:** 2018-04-01

**Authors:** Rashed Alremeithi, Mohammed A. Meetani, Anas A. Alaidaros, Adnan Lanjawi, Khalid Alsumaiti

**Affiliations:** ^1^Chemistry Department, United Arab Emirates University, P.O. Box 15551, Al-Ain, UAE; ^2^General Department of Forensic Science and Criminology, Dubai Police, Dubai, UAE

## Abstract

Development and validation of sensitive and selective method for enantioseparation and quantitation of synthetic cathinones is reported using GC-MS triple quadrupole mass spectrometry with negative chemical ionization (NCI) mode. Indirect chiral separation of thirty-six synthetic cathinone compounds has been achieved by using an optically pure chiral derivatizing agent (CDA) called (*S*)-(−)-*N*-(trifluoroacetyl)pyrrolidine-2-carbonyl chloride (L-TPC), which converts cathinone enantiomers into diastereoisomers that can be separated on achiral columns. As a result of using Ultra Inert 60 m column and performing slow heating rate (2°C/min) on the GC oven, an observed enhancement in enantiomer peak resolution has been achieved. An internal standard, (+)-cathinone, was used for quantitation of synthetic cathinones. Method validation in terms of linearities and sensitivity in terms of limits of detection (LODs), limits of quantitation (LOQs), recoveries, and reproducibilities has been obtained for fourteen selected compounds that examined simultaneously as a mixture after being spiked in urine and plasma. It was found that the LOD of the fourteen synthetic cathinones in urine was in the range of 0.26–0.76 *µ*g/L, and in plasma, it was in the range of 0.26–0.34 *µ*g/L. While the LOQ of the mixture in urine was in the range of 0.86–2.34 *µ*g/L, and in plasma, it was in the range of 0.89–1.12 *µ*g/L. Unlike the electron impact (EI) ion source, NCI showed better sensitivity by two orders of magnitude by comparing the obtained results with the recently published reports for quantitative analysis and enantioseparation of synthetic cathinones.

## 1. Introduction

From the beginning of the new century till now, governments and forensic science specialists are suffering from a nightmare called new designer substances (NDS), which comprise a risk in society that is growing up day by day. Presently, the latest version of NDS is called “bath salts,” and they overrun the drug of abuse market. Bath salts are a group of central nervous system stimulants that consists mainly of synthetic cathinone derivatives [1]. In nature, cathinone (*β*-keto amphetamine) exists in the leaves of the *Catha edulis* plant, which can be found easily in the region of northeast Africa and the Arabian Peninsula [2]. However, scientists have synthesized cathinones in laboratory when the Germans and the French chemists synthesized methcathinone for the first time in the late 1920s [3]. During the 1930s and 1940s, methcathinone was available in pharmacological markets as an appetite suppressant and antidepressant medicine [4]. Methcathinone abuse spread to the USA at 1991, and as a result of that, it was included in the UN Convention on Psychotropic Substances [5]. In the meantime, drug dealers were looking for new strategies to sell their products and they found it by the “novel psychoactive substances,” drugs which contain at least one chemical substance that has similar biological effects as of illegal drugs. For instance, “Explosion” is the trade name of the synthetic cathinone methylone, which emerged for sale in Japan and Netherlands via the Internet in 2004 [6]. In 2007, 4-methyl methcathinone (mephedrone) became one of the most commonly abused drugs in Europe [6]. Thus, concerns about the abuse of novel psychoactive substances especially cathinone-related derivatives grew up in Europe which gave rise to ban of cathinone derivatives in April 2010 by the UK government and by the European Monitoring Centre for Drugs and Drug Addiction (EMCDDA) [7]. Despite all the actions taken by legal authorities, an intense attention by drug dealers has been put on the synthesis of new generations of synthetic cathinone derivatives.

In order to obviate the abuse risks of these psychoactive stimulants, focused studies should be carried out on the neuropharmacological properties of the active compounds. This can be accomplished by separating the enantiomers using a selective and sensitive method. However, the current separation and detection methods are not completely effective; therefore, a new separation and detection method is reported in this work. In nature, cathinone exists as a racemic mixture that contains one chiral center which means that it has two enantiomers and commonly one of them will have greater psychological effect in human biological system than the other enantiomer [8]. For example, it has been found that the stimulating effect of (*S*)-methcathinone is higher than (*R*)-methcathinone [8]. However, the literature limitation of the pharmacological data for the new cathinone derivatives racemates lets researchers assume that the case for most phenylalkylamine compounds will be similar to methcathinone. As a result of that, enantioseparation of chiral synthetic cathinones became an attractive and promised field of research where the use of major separation techniques took place such as gas chromatography [8, 9], high-performance liquid chromatography (HPLC) [10–15], and capillary electrophoresis (CE) [15–22].

Generally, the principle of chiral separation can be summarized by two different techniques: direct and indirect chiral separation. The previously mentioned separation techniques can be satisfied by applying chiral separation principles. The use of direct separation technique for the enantiomers implies the use of chiral selector which can be either immobilized on the stationary phase of the column or dissolved in the mobile phase of the separation system as in the case for some HPLC and CE chiral methods [23]. However, indirect chiral separation can be achieved by converting enantiomers to diastereoisomers via derivatization reaction of the targeted compounds with optically pure chiral derivatizing agents (CDAs) [9]. Moreover, the resulted diastereoisomers could be separated on achiral stationary phase column in GC or HPLC system. (*S*)-(−)-*N*-(trifluoroacetyl)pyrrolidine-2-carbonyl chloride (L-TPC) is one of the well-known CDAs that is readily available in chemical market and shows impressive results in chiral separation of the phenylalkylamines mainly on GCMS after derivatization reaction [8, 9, 24].

In the literature, only few papers have discussed the chiral separation of L-TPC cathinone derivatives by using GC-EI-MS [8, 9, 25]. Electron impact (EI) is the most preferable ionization source in GC-MS, which provides characteristic and reproducible mass spectrum for each compound. EI is considered as a hard ionization technique which provides mass spectra that are rich with low mass fragments and usually the molecular ion peak is absent [26]. Recently, a short communication on the analysis of twenty-nine synthetic cathinones in GC-MS/MS with positive chemical ionization (PCI) mode has been reported [27]. However, no quantitative assessment was given for these compounds in biological fluids. Unlike EI, determination of molecular weight and structure elucidation can be carried out through the use of chemical ionization source coupled with tandem mass spectrometry [27]. Furthermore, when the investigated compounds are electronegative moieties, the use of NCI mode can dramatically improve the sensitivity of the targeted compounds [28]. In NCI, negative ions are mainly formed by capturing thermal electrons (low-energy electrons with nearly 0–2 eV), and this ionization process is called resonance electron capture. The electrons are produced from the filament and lose their energy by collision and ionization of the reagent gas molecules. If electrons have enough energy (2–15 eV) to break up molecules, fragmentation occurs and this ionization process is called dissociative electron capture. NCI is highly sensitive and selective for compounds with a positive electron affinity. It is a soft ionization method, like PCI, so a NCI spectrum is relatively simple [29].

There are no reports in the literature that discuss the use of GC-MS in negative chemical ionization mode for quantitative analysis of synthetic cathinones. The electrons emitted from a filament lose their energy to become thermal electrons by collision with reagent gas and ionization of reagent gas molecules. Nearly, 0 eV electrons are captured by molecules so that molecular ions are produced (resonance electron capture). If electrons have enough energy to break up molecules, fragmentation occurs (dissociative electron capture) [29].

**Scheme 1:**
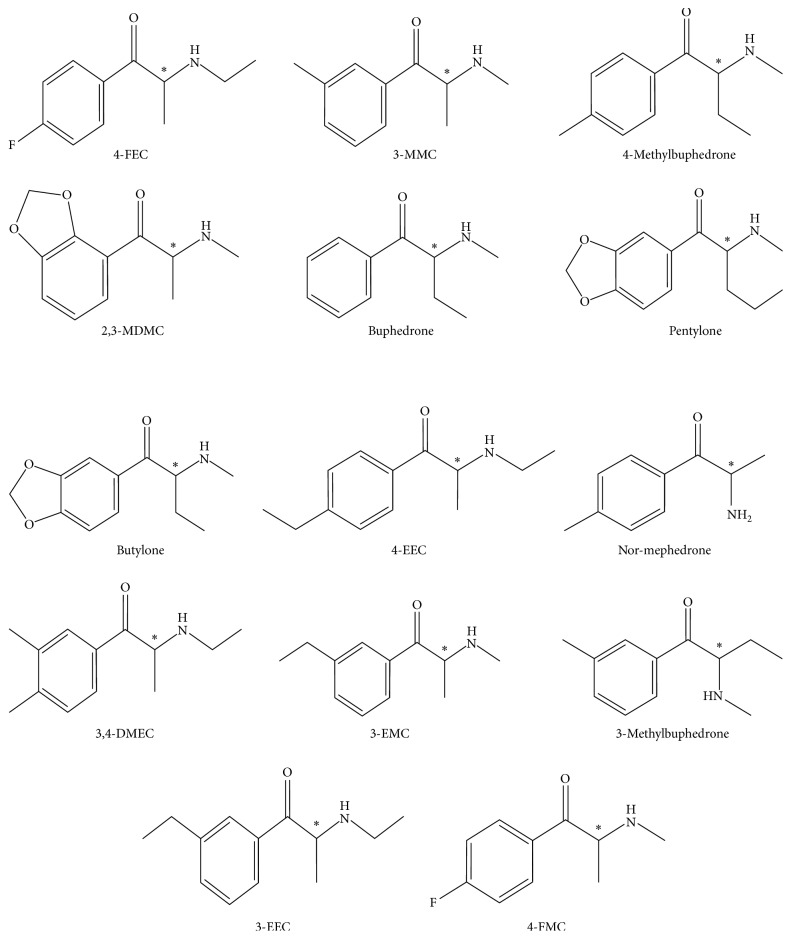


In this work, a sensitive and selective GC-NCI-MS method has been developed to analyze thirty-six synthetic cathinone compounds after their conversion into diastereoisomers through the derivatization reaction with L-TPC. Quantitative analysis of spiked urine and plasma samples was conducted for fourteen of these synthetic cathinones (Scheme 1), which were analyzed in one mixture simultaneously. The method validation was performed on spiked biological samples and found to produce complete separation of the synthetic cathinone enantiomers on achiral capillary GC column in addition to sensitive detection of low concentrations in the *µ*g/L range better than the previous reported methods that use EI and positive CI ionization mass spectrometry.

## 2. Experimental

### 2.1. Chromatographic Conditions

Chromatographic separation was performed on an Agilent 7890A GC coupled to an Agilent 7000 Triple Quad mass selective detector. A commercially available 60 m HP-5MS Ultra Inert capillary column, with 0.25 mm inner diameter and a 0.25 *µ*m film thickness was used as the stationary phase. Chemical ionization (CI) with methane gas (40%, 2.0 mL/min) was employed in the negative ion mode at a voltage of 70 eV. Helium was used as the carrier gas at a constant flow rate of 0.8 mL/min. Injection of 3 *µ*l of sample solution was performed automatically in splitless mode. The injector and GC-MS interface temperatures were set at 250 and 280°C, respectively. Data collection was performed in selected ion monitoring (SIM) mode with the selected fragment ions as shown in Table 1, starting at 30 min after injection. The column temperature program was as follows: starting at 160°C and then holding for 5 min, followed by subsequent heating to 260°C at a heating rate of 2°C/min. The final temperature was held at 260°C for 10 min.

### 2.2. Chemicals and Reagents

All chemicals were of analytical grade. Ethyl acetate, acetic acid, methanol, 2-propanol, ammonium hydroxide, dichloromethane, 0.1 M solution of (*S*)-(−)-*N*-(trifluoroacetyl)pyrrolidine-2-carbonyl chloride (L-TPC) with an enantiomer excess (ee) of 97% (according to the supplier's specification) in methylene chloride, anhydrous sodium sulfate, and sodium phosphate were obtained from Sigma-Aldrich Chemicals (St. Louis, MO, USA). Potassium carbonate was obtained from VWR (Darmstadt, Germany). Doubly deionized water was obtained from Ultra-Pure Millipore system (MS, USA). All chemicals shown in Table 2 were purchased from Cayman Chemicals (Michigan, USA) and were provided as racemic mixtures for individual cathinones (99% purity).

### 2.3. Sample Preparation

#### 2.3.1. Samples

This investigation conforms to the UAE community guidelines for the use of humans in experiments. The Human Ethics Committee at the Dubai Police approved this study. Blood and urine samples were collected by Dubai Police with the consent of the subjects.

#### 2.3.2. Solid-Phase Extraction (SPE) of Spiked Urine and Plasma Samples

**Scheme 2:**
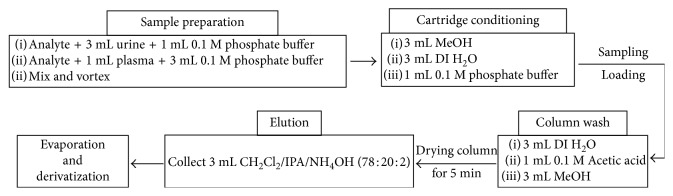


SPE was carried out using “Zymark rapid trace” SPE workstation (Artisan Technology Group, Champaign, IL, USA), and the column was 200MG clean screen CSDAU203 from FluoroChem (Hadfield, UK). Urine samples were diluted in 1 : 2 ratio with doubly deionized water. Diluted urine (3 mL) was spiked with certain concentration of synthetic cathinones and 20 *µ*g/L of IS ((+)-cathinone) in addition to 1 mL of 0.1 M phosphate buffer (pH 6). For the spiking of plasma samples, 1 mL of plasma was spiked with certain concentration of synthetic cathinones and 50 ppm of IS ((+)-cathinone) was added in addition to 3 mL of 0.1 M phosphate buffer (pH 6). Sample was shaken thoroughly for 30 s. The SPE cartridge was conditioned by adding 3 mL of methanol, and the same volume of deionized water was used with 1 mL of 0.1 M phosphate buffer. After that, the spiked urine or plasma sample was loaded to the cartridge and later the cartridge was washed by 3 mL of methanol followed by 3 mL of deionized water, and finally, 1 mL of 0.1 M acetic acid was added. The column was left for drying for 5 min. Finally, 3 mL of the eluate was collected and evaporated to dryness under nitrogen gas. Solid-phase extraction procedure is summarized in Scheme 2.

#### 2.3.3. Derivatization Step

**Scheme 3:**
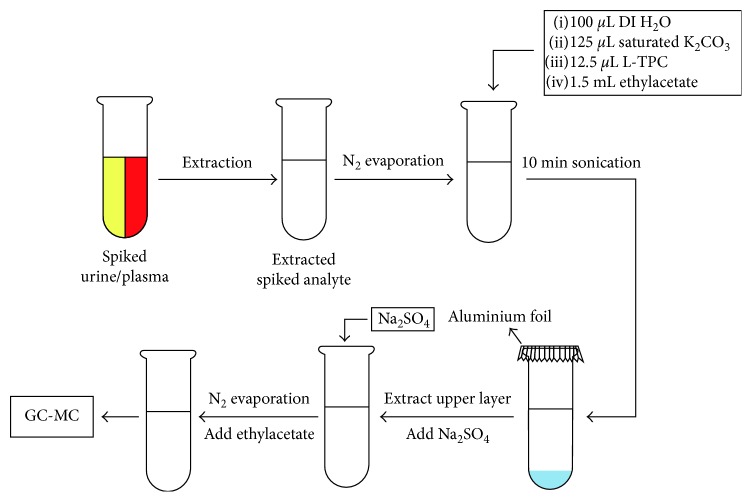


For the analysis of pure and spiked samples, evaporation step is necessary before derivatization reaction can take place. After the evaporation is done, 100 *µ*l of deionized water was transferred into a glass test tube containing the pure sample together with 125 *µ*l of a saturated aqueous solution of potassium carbonate, 1.5 mL of ethyl acetate, and 12.5 *µ*l of L-TPC. For the analysis of spiked urine and plasma, 50 *µ*l of L-TPC was used. The mixture was covered and stirred for 10 min at room temperature. Afterwards, the upper layer was transferred to a new test tube and dried over anhydrous sodium sulfate. The dried solution was evaporated to completion under a gentle nitrogen stream. The remaining L-TPC derivative was reconstituted in certain amount of ethyl acetate—depending on concentration—prior to injection in GC-MS instrument.  Scheme 3 summarizes the L-TPC derivatization process of the synthetic cathinones.

### 2.4. Method Validation

The combination of SPE with L-TC derivatization proved to be useful for the determination of synthetic cathinones in urine and plasma samples, as no interferences from endogenous and exogenous compounds were observed. During the method validation, various parameters of the method such as linearity, sensitivity, accuracy, recovery, and reproducibility were evaluated according to international criteria.

## 3. Results

**Figure 1:**
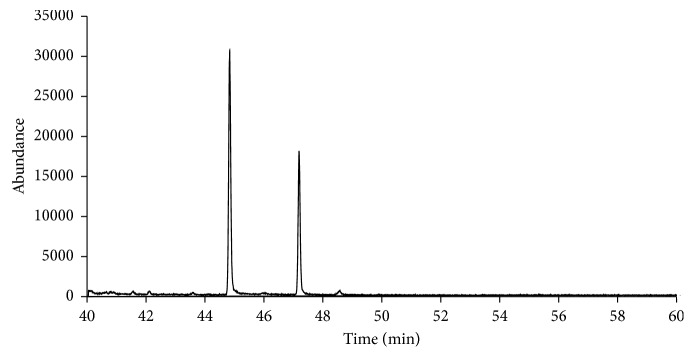
Gas chromatogram for separation of the *R* and *S* enantiomers of Nor-mephedrone drug in methanol after derivatization with L-TPC.

The indirect chiral separation method that has been developed is based on the conversion of synthetic cathinones to L-TPC derivatives. A normal (or achiral) stationary phase capillary column has been used for the separation of the resulting diastereomers due to their different chemical and physical properties. The primary and secondary amine cathinones react with the derivatization reagent L-TPC in the presence of sodium carbonate, and the amidation reaction occurs between the acid chloride in L-TPC and the amine group of the target analytes. The gas chromatogram in Figure 1 shows the separation of the (*R*) and (*S*) enantiomers of nor-mephedrone drug after derivatization with L-TPC. Table 2 shows the retention times, resolution, and selectivity factors of the separated enantiomers of all the studied synthetic cathinones. All compounds in Table 2 were analyzed individually on GC-MS using SIM mode, after going through the derivatization step.

**Figure 2:**
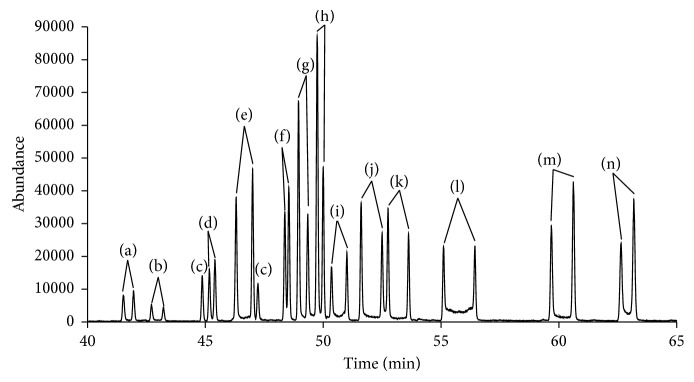
Total ion current chromatogram (TIC) of the simultaneous chiral separation of 14 synthetic cathinone compounds spiked in urine and separated as the following L-TPC derivatives: (a) 4-FMC, (b) 4-FEC, (c) nor-mephedrone, (d) buphedrone, (e) 3-MMC, (f) 3-methylbuphedrone, (g) 4-methylbuphedrone, (h) 3-EMC, (i) 3-EEC, (j) 4-EEC, (k) 3,4-DMEC, (l) 2,3-MDMC, (m) butylone, and (n) pentylone.

**Figure 3:**
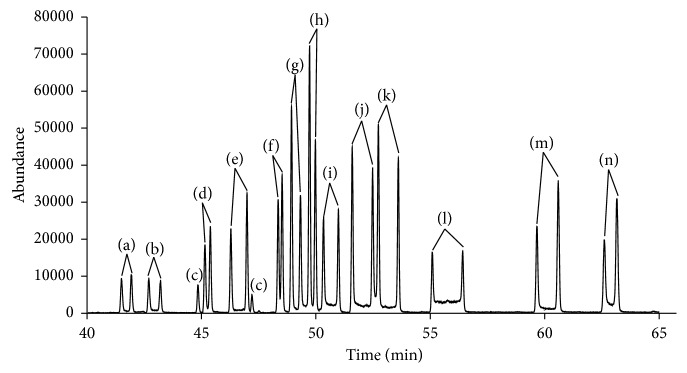
Total ion current chromatogram (TIC) of the simultaneous chiral separation of 14 synthetic cathinone compounds spiked in plasma and separated as the following L-TPC derivatives: (a) 4-FMC, (b) 4-FEC, (c) nor-mephedrone, (d) buphendrone, (e) 3-MMC, (f) 3-methylbuphedrone, (g) 4-methylbuphedrone, (h) 3-EMC, (i) 3-EEC, (j) 4-EEC, (k) 3,4-DMEC, (l) 2,3-MDMC, (m) butylone, and (n) pentylone.

Figures 2 and 3 show the total ion current chromatogram of the fourteen synthetic cathinones spiked in urine and plasma, respectively. The resulted enantiomer peaks were well separated with good peak resolution. To our knowledge, this is the first example in the literature that demonstrates the separation of fourteen pairs of L-TPC cathinone derivatives in one run analysis of these compounds in complex matrices of urine and plasma.

Validation of the developed method was performed on spiked mixtures successfully. Linearity of the calibration curves, method sensitivity in terms of LOD and LOQ, and recoveries in addition to interday and intraday reproducibilities were collected and summarized in Tables 3–5.

The calibration curves for the fourteen synthetic cathinones derivatives were found to be linear within the tested range of 1 to 100 *µ*g/L in urine and in plasma with mean regression coefficients (*R*^2^; *n*=3) higher than 0.99. The regression coefficients and the LOD and LOQ values for the two enantiomers of the synthetic cathinone compounds in the mixture that spiked in urine and plasma are reported in Table 3. Three different concentration levels were tested for each enantiomer of these compounds (20, 60 and 100 *µ*g/L) in order to ensure the reproducibility and to provide the recovery study of the new method. The interday and intraday reproducibilities of the cathinones mixture of urine and plasma matrices are shown in Table 4. Moreover, percent error evaluation has been done for the spiked mixture to obtain the recovery studies which are summarized in Table 5.

## 4. Discussion

L-TPC is considered as a chiral derivatizing agent which can react with the primary and secondary amine enantiomers of synthetic cathinones producing two corresponding diastereomers. As a result of the differences in stereochemistry and stability of the formed diastereoisomers, the enantioseparation can occur on achiral stationary phase with different resolutions of product compounds [24, 30]. In this study, chiral separation of 36 racemic mixtures of synthetic cathinone derivatives was carried out: fourteen of them were selected in the spiked mixtures, and each enantiomer was quantitated in urine and plasma as shown in the example of nor-mephedrone in Figure 1. However, the enantioseparations that were obtained showed that there are differences in peak areas for the most resulted diastereoisomers. Mohr et al. assumed that the reason of inequality in the formed peaks is due to (i) racemization of L-TPC during the derivatization reaction, (ii) kinetic resolution of the two enantiomers, and (iii) the difference in diastereoisomers' yields which were explained in terms of keto-enol tautomerization of the analytes. Moreover, the main reason for enantiomer peak inequality is related to the tested compounds themselves [8].

Interestingly, the fourteen spiked synthetic cathinone derivatives were separated simultaneously in one chromatogram since they have different retention times in the new developed method as shown in Figures 2 and 3, respectively. Moreover, enhancement of the resolution of enantiomers' peaks has been accomplished by using a slow heating rate of 2°C/min in the chromatographic method. Also, the use of Ultra Inert column helped in minimizing the overlap of the two adjacent peaks of the enantiomers. Chemical ionization conditions have allowed better detection of molecular ion peaks (M-H^−^) and minimized the extensive fragmentation of the targeted analytes.

Construction of calibration curves was done for the diastereoisomers based on the peak areas of the following concentration levels: 1, 5, 10, 20, 40, 60, 80, and 100 *µ*g/L. Regression values of the correlation coefficient confirm the good linearity of the four calibration lines. In order to correct for the loss of analyte during sample inlet or sample preparation, (+)-cathinone has been used as IS as it has a similar structure to synthetic cathinones and shows a good stability. The correlation coefficient (*R*^2^) values were calculated for the mixture components, and they were found to be higher than 0.99 in all cases as shown in Table 3. Additionally, the LODs and LOQs were calculated according to the IUPAC method and are reported in Table 3. The reported values of LODs and LOQs for the synthetic cathinones in this study were in the *µ*g/L range due to the high sensitivity of the analytical technique (GC-NCI-MS). The high mobility electrons that have low mass and energy produced during the NCI process are responsible for enhanced sensitivity when used for a suitably electrophilic compound compared to PCI and EI [29]. The LOD in urine was in the range of 0.26–0.76 *µ*g/L, and in plasma, it was in the range of 0.26–0.34 *µ*g/L. While the LOQ in urine was in the range of 0.89–2.34 *µ*g/L, and in plasma, it was in the range of 0.89–1.12 *µ*g/L (as shown in Table 3).

Three different concentration levels were chosen to test the interday and intraday reproducibility measurements of the synthetic cathinone compounds mixture of urine and plasma as shown in Table 3. In fact, good reproducibility and repeatability were established using the new developed method since most of the coefficients of variance values were below 15% in both urine and plasma matrices for measurements done on the same day or on two different days. In comparison, between spiked urine and spiked plasma samples, urine samples were more reproducible than spiked plasma samples because of the competition between analyte and blood interferences unlike spiked urine samples where the urine was diluted with deionized water prior to the spiking step. Moreover, the presence of proteins and other interferences in plasma can cause difficulty in solid-phase extraction processes and can also create a competition between the targeted analyte and unneeded interferences which will lead to variation in spiked plasma results [31].

SPE efficiency was studied by percent error calculations for the spiked mixture at the following concentration levels: 20, 60, and 100 *µ*g/L. The calculated values in recovery studies were within the acceptable range.

By comparing the results of the GC-EI-MS method recently reported for some of these synthetic cathinones [25] and the current study results using GC-NCI-MS, the latter has shown an enhancement of sensitivity by a magnitude of two orders. The high sensitivity of NCI is due to the low mass and high mobility of the secondary or thermal electrons (low-energy electrons) produced under the CI high pressure conditions in the presence of methane reagent gas, which is responsible for the enhancement factor by nearly 100 times in the sensitivity of NCI compared to that of positive EI or CI for a suitably electrophilic compound [29].

## 5. Conclusion

Indirect chiral separation of synthetic cathinones after derivatization with trifluoroacetyl-l-prolyl chloride (L-TPC) was achieved using a new developed method of GC-NCI-MS in SIM mode, which provided high sensitivity and selectivity for the separation and quantitation of the targeted compounds. The use of 60 m HP-5MS Ultra Inert capillary column helps to separate more than thirty-six compounds of synthetic cathinones to their diastereomers. NCI has shown to be an effective ionization method for these cathinones and resulted in lower detection limits when compared to previous reports. A mixture of fourteen cathinone derivatives that were spiked in urine and plasma was separated in one chromatogram simultaneously. For each enantiomer peak in the cathinone mixture chromatogram, calibration curve was constructed using the following concentration levels: 1, 5, 10, 20, 40, 60, 80, and 100 *µ*g/L. The developed method was validated in terms of linearities, LOD, LOQ, reproducibilities, and recoveries for all the tested mixtures.

## Figures and Tables

**Table 1:** Time segments table with selected ions used in SIM mode for the analysis of cathinone mixture.

Compound name	Abbreviation	Time	Mass
(+)-Cathinone	—	39.00–41.00	189^*∗*^, 209, 342
4-Fluoromethcathinone	4-FMC	41.00–42.50	153, 223^*∗*^, 374
4-Fluoroethcathinone	4-FEC	42.50–44.70	167^*∗*^, 237, 388
Nor-mephedrone	—	44.70–45.06	189, 209^*∗*^, 356
Buphedrone	—	45.06–47.13	153^*∗*^, 223, 370
3-Methylmethcathinone	3-MMC		
Nor-mephedrone		47.13–48.00	189^*∗*^, 209, 356
3-Methylbuphedrone	—	48.00–50.15	153^*∗*^, 223, 384
4-Methylbuphedrone	—		
3-Ethylmethcathinone	3-EMC		
3-Ethylethcathinone	3-EEC	50.15–54.00	167^*∗*^, 237, 398
4-Ethylethcathinone	4-EEC		
3,4-Dimethylethcathinone	3,4-DMEC		
2,3-Methylenedioxymethcathinone	2,3-MDMC	54.00–59.00	153^*∗*^, 223, 400
Butylone	—	59.00–62.00	153, 223^*∗*^, 414
Pentylone	—	62.00–65.00	156, 223^*∗*^, 428

^*∗*^The quantifier mass.

**Table 2:** List of the 36 cathinone-related compounds and their synonyms, in addition to the retention times of the separated two diastereoisomers for each compound analyzed by GC-MS using SIM mode.

	Name	Synonyms	Time (min)		FWHM		Resolution	Selectivity factor (*α*)
			*t*_*R*1_	*t*_*R*2_	*P*1	*P*2		
1	2-Methoxymethcathinone	2-MeOMC	48.98	49.98	0.061	0.062	9.59	1.03
2	3-Fluoroethcathinone	3-FEC	43	43.3	0.066	0.068	2.64	1.01
3	4-Fluoroethcathinone	4-FEC	42.7	43.2	0.084	0.086	3.47	1.01
4	2,3-Methylenedioxymethcathinone	2,3-MDMC	55.1	56.4	0.078	0.075	10.03	1.03
5	2-Methylmethcathinone	2-MMC	45.1	46.2	0.065	0.07	9.61	1.03
6	Nor-mephedrone	—	44.9	47.2	0.078	0.081	17.07	1.06
7	4-Ethylethcathinone	4-EEC	51.6	52.5	0.075	0.076	7.03	1.02
8	3,4-Dimethylethcathinone	3,4-DMEC	52.8	53.6	0.072	0.072	6.56	1.02
9	2-Ethylmethcathinone	2-EMC	47.5	48.6	0.064	0.07	9.69	1.03
10	3-Methoxymethcathinone	3-MeOMC	51.4	51.7	0.059	0.06	2.97	1.01
11	2-Fluoromethcathinone	2-FMC	41.9	43	0.073	0.076	8.71	1.03
12	4-Ethylmethcathinone	4-EMC	50.5	51.7	0.061	0.061	11.61	1.03
13	3-Ethylethcathinone	3-EEC	50.4	51	0.078	0.072	4.72	1.01
14	4-Methylbuphedrone	—	48.96	49.4	0.074	0.078	3.42	1.01
15	2,3-Dimethylmethcathinone	2,3-DMMC	49.7	51.1	0.062	0.069	12.61	1.03
16	3-Ethylmethcathinone	3-EMC	49.8	50	0.078	0.074	1.55	1.00
17	3-Fluoromethcathinone	3-FMC	41.7	41.98	0.07	0.065	2.45	1.01
18	4-Fluoromethcathinone	4-FMC	41.5	41.96	0.092	0.084	3.08	1.01
19	2-Methylethcathinone	2-MEC	46.3	47.6	0.073	0.068	10.88	1.03
20	Buphedrone	—	45.2	45.4	0.079	0.08	1.48	1.01
21	4-Methyl-*α*-ethylaminobutiophenone	—	49.7	50.3	0.061	0.061	5.80	1.01
22	Pentedrone	—	47.6	47.7	0.057	0.053	1.07	1.00
23	Butylone	—	59.7	60.6	0.091	0.091	5.84	1.02
24	Pentylone	—	62.6	63.2	0.105	0.103	3.40	1.01
25	4-Methylethcathinone	4-MEC	48	49.2	0.058	0.063	11.70	1.03
26	Ethcathinone	—	44.2	44.9	0.067	0.067	6.16	1.02
27	3-Methylmethcathinone	3-MMC	46.3	47	0.078	0.077	5.33	1.02
28	4-Bromomethcathinone	4-BMC	53.96	54.3	0.069	0.068	2.93	1.01
29	3-Bromomethcathinone	3-BMC	42.9	43.6	0.090	0.085	4.72	1.02
30	2,4-Dimethylmethcathinone	2,4-DMMC	48.5	49.96	0.063	0.063	13.67	1.04
31	2,4-Dimethylethcathinone	2,4-DMEC	49.8	51.2	0.065	0.071	12.15	1.03
32	3,4-Methylenedioxy-*N*-ethylcathinone	Ethylone	58.6	59.9	0.075	0.079	9.96	1.03
33	3-Methylbuphedrone	—	48.4	48.5	0.074	0.074	0.80	1.00
34	*N*-ethylbuphedrone	NEB	45.9	46.1	0.061	0.061	1.93	1.01
35	2,3-Pentylone isomer	—	59.1	59.9	0.058	0.07	7.37	1.02
36	3-Methylethcathinone	3-MEC	47.4	48.1	0.063	0.061	6.66	1.02

**Table 3:** Results for fourteen cathinone-related compounds spiked in plasma and urine including linearity coefficient, *R*^2^ values, limits of detection, and limits of quantitation for the two enantiomers of each compound.

		Plasma						Urine					
		*R* ^2^		LOQ (*µ*g/L)		LOD (*µ*g/L)		*R* ^2^		LOQ (*µ*g/L)		LOD (*µ*g/L)	
		E1	E2	E1	E2	E1	E2	E1	E2	E1	E2	E1	E2
1	4-FMC	0.9905	0.9962	0.957 ± 0.14	0.924 ± 0.12	0.29 ± 0.08	0.28 ± 0.08	0.9903	0.9900	1.09 ± 0.11	1.06 ± 0.11	0.33 ± 0.08	0.32 ± 0.08
2	4-FEC	0.9908	0.9902	1.023 ± 0.15	1.122 ± 0.13	0.31 ± 0.08	0.34 ± 0.08	0.9959	0.9914	1.82 ± 0.16	2.34 ± 0.16	0.55 ± 0.1	0.71 ± 0.1
3	Nor-mephedrone	0.9902	0.9931	0.891 ± 0.09	1.056 ± 0.14	0.27 ± 0.08	0.32 ± 0.08	0.9949	0.9926	2.15 ± 0.15	2.51 ± 0.16	0.65 ± 0.1	0.76 ± 0.1
4	Buphedrone	0.9933	0.9907	0.891 ± 0.09	0.858 ± 0.08	0.27 ± 0.08	0.26 ± 0.08	0.9931	0.9922	0.96 ± 0.14	0.92 ± 0.10	0.29 ± 0.07	0.28 ± 0.07
5	3-MMC	0.9904	0.9903	0.891 ± 0.09	0.858 ± 0.08	0.27 ± 0.08	0.26 ± 0.08	0.9944	0.9957	1.02 ± 0.15	0.92 ± 0.09	0.31 ± 0.08	0.28 ± 0.07
6	3-Methylbuphedrone	0.9902	0.9914	0.99 ± 0.11	1.023 ± 0.15	0.3 ± 0.08	0.31 ± 0.08	0.9941	0.9931	1.02 ± 0.15	0.99 ± 0.11	0.31 ± 0.07	0.3 ± 0.07
7	4-Methylbuphedrone	0.9910	0.9919	1.056 ± 0.10	1.056 ± 0.10	0.32 ± 0.08	0.32 ± 0.08	0.9902	0.9919	1.12 ± 0.13	1.09 ± 0.11	0.34 ± 0.09	0.33 ± 0.08
8	3-EMC	0.9945	0.9915	1.155 ± 0.11	1.089 ± 0.10	0.35 ± 0.08	0.33 ± 0.08	0.9901	0.9906	1.12 ± 0.11	1.06 ± 0.11	0.34 ± 0.08	0.32 ± 0.08
9	3-EEC	0.9952	0.9905	1.023 ± 0.11	0.957 ± 0.14	0.31 ± 0.08	0.29 ± 0.08	0.9980	0.9921	1.02 ± 0.15	1.09 ± 0.10	0.31 ± 0.07	0.33 ± 0.08
10	4-EEC	0.9942	0.9907	0.957 ± 0.14	0.858 ± 0.10	0.29 ± 0.08	0.26 ± 0.08	0.9907	0.9901	1.09 ± 0.10	1.12 ± 0.11	0.33 ± 0.08	0.34 ± 0.07
11	3,4-DMEC	0.9904	0.9909	0.99 ± 0.10	1.056 ± 0.10	0.3 ± 0.08	0.32 ± 0.08	0.9924	0.9912	1.12 ± 0.11	1.39 ± 0.11	0.34 ± 0.08	0.42 ± 0.09
12	2,3-MDMC	0.9900	0.9918	1.122 ± 0.10	1.089 ± 0.10	0.34 ± 0.08	0.33 ± 0.08	0.9927	0.9916	1.35 ± 0.13	1.35 ± 0.11	0.41 ± 0.09	0.41 ± 0.09
13	Butylone	0.9950	0.9920	0.924 ± 0.09	0.957 ± 0.14	0.28 ± 0.08	0.29 ± 0.08	0.9920	0.9908	0.86 ± 0.11	0.89 ± 0.09	0.26 ± 0.08	0.27 ± 0.07
14	Pentylone	0.9940	0.9969	0.858 ± 0.09	0.957 ± 0.14	0.26 ± 0.08	0.29 ± 0.08	0.9923	0.9901	0.96 ± 0.10	0.86 ± 0.11	0.29 ± 0.07	0.26 ± 0.08

**Table 4:** Interday and intraday reproducibility results in terms of coefficient of variance for fourteen cathinone-related compounds spiked in urine and plasma at three different concentration levels for the two enantiomers of each compound.

			CV% intraday						CV% interday					
			20 *µ*g/L		60 *µ*g/L		100 *µ*g/L		20 *µ*g/L		60 *µ*g/L		100 *µ*g/L	
			E1	E2	E1	E2	E1	E2	E1	E2	E1	E2	E1	E2
1	4-FMC	U	3.61	3.11	1.04	1.79	0.41	1.33	10.26	11.85	10.28	12.02	8.14	7.6
		P	6	4.79	2.59	5.36	2.26	2.59	11.01	10.6	10.99	13.3	10.95	12.35
2	4-FEC	U	2.22	5.32	1.75	1.53	2.03	1.69	5.67	10.9	14.69	15.74	15.8	16.13
		P	10.57	11.65	5.31	3.89	1.69	2.21	7.44	8.43	7.59	11.25	6.65	7.76
3	Nor-mephedrone	U	0.7	1.01	2.13	1.64	2	2.17	3.07	4.57	3.5	4.43	1.75	4.91
		P	7.59	7.95	3.07	3.51	2.79	6.97	6.01	7.1	14.13	6.48	14.3	14.21
4	Buphedrone	U	1.42	1.6	1.89	2.28	2.12	1.17	5.6	14.08	15.31	16.91	16.62	15.29
		P	11.13	10.88	3.37	6.27	1.32	3.14	8.04	8.49	13.39	14.26	5	8.32
5	3-MMC	U	3.68	2.03	0.93	1.66	0.39	1.34	12.27	9.02	1.72	2.41	0.82	1.29
		P	9.74	12.68	4.55	6.19	2.51	3.69	8.42	9.85	4.03	4.78	16.3	16.97
6	3-Methylbuphedrone	U	1.36	3.07	2.06	4.48	0.93	1.09	5.32	14.73	6.73	11.96	10.04	9.44
		P	10.58	11.42	5.04	5.44	1.19	3.58	7	7.9	10.55	15.34	13.67	14.31
7	4-Methylbuphedrone	U	1.25	2.16	0.35	0.75	0.68	1.14	3.73	11.28	9.25	2.04	10.22	9.93
		P	9.77	11.67	5.41	4.49	2.42	2.82	9.25	11.85	19.55	6.83	18.03	19.59
8	3-EMC	U	2.38	2.57	0.63	2.1	0.74	1.69	14.53	14.81	13.18	3.51	7.62	10.98
		P	11.36	8.27	5.41	4.55	3.01	3.16	11.35	9.79	19.65	7.13	19.04	17.92
9	3-EEC	U	3.22	6.13	1.13	3.6	1.71	2.13	13.29	15.44	8.42	10.94	3.27	4.1
		P	8.81	12.64	4.3	5.28	3.15	3.71	6.62	9.27	10.19	12.67	14.15	14.82
10	4-EEC	U	3.45	1.85	0.93	2.17	0.84	0.82	14.48	11.31	9.31	12.68	7.19	1.8
		P	11.48	10.53	6.12	4.48	4.24	3.59	8.31	9.46	19.81	19.9	10.97	15.42
11	3,4-DMEC	U	3.7	3.5	1.97	1.02	3.44	2.16	16.54	14.77	10.92	14.49	3.07	2.62
		P	12.11	13.14	4.5	6.31	3.26	3.06	9.44	11.05	18.98	20.31	9.66	15.7
12	2,3-MDMC	U	2.07	3.24	3.16	1.43	4.11	5.56	5.5	4.43	13.57	14.03	5.74	6.6
		P	10.94	7.12	2.74	4.02	7.24	7.13	9.2	5.11	3.41	4.46	19.77	19.35
13	Butylone	U	5.14	6.26	1.36	0.5	0.85	0.88	12.57	14.11	14.56	14.9	1.25	1.56
		P	10.78	12.89	4.97	6.67	2.63	3.25	11.42	13.6	3.66	7.62	9.06	9.14
14	Pentylone	U	7.1	1.34	2.59	1.84	1.83	0.19	12.98	5.99	12.77	14.03	2.29	3.86
		P	10.2	18.64	3.74	5.93	2	2.06	10.29	19.91	9.77	6.67	5.01	6.59

U: urine; P: plasma; CV: coefficient of variance; E1: enantiomer 1; E2: enantiomer 2.

**Table 5:** Recovery measurements expressed in percent errors for three different concentrations of the cathinone-related compounds spiked in urine matrix.

		Plasma (error %)						Urine (error %)					
		20 *µ*g/L		60 *µ*g/L		100 *µ*g/L		20 *µ*g/L		60 *µ*g/L		100 *µ*g/L	
		E1	E2	E1	E2	E1	E2	E1	E2	E1	E2	E1	E2
1	4-FMC	2.63	5.32	2.48	0.62	2.45	0.26	0.8	10.57	9.49	8.47	1.24	0.68
2	4-FEC	0.17	4.89	5.8	3.1	2.19	1.6	9.28	5.31	2.38	9.21	0.1	0.61
3	Nor-mephedrone	5.64	1.94	3.25	7.64	1.69	2.64	8.67	7.48	1.82	4.48	1.71	0.69
4	Buphedrone	3.18	5.94	0.79	3.78	0.06	1.71	9.12	5.58	4.84	4.88	1.9	0.72
5	3-MMC	1.03	1.47	0.87	0.55	0.74	1.4	3.91	3.02	4.91	3.54	0.25	0.91
6	3-Methylbuphedrone	1.26	7.1	6.41	9.61	4.03	1.71	3.71	9.13	4.09	0.15	1.54	1.77
7	4-Methylbuphedrone	0.69	5.64	1.77	0.73	2.56	1.9	1.15	0.42	0.97	2.54	2.3	4.01
8	3-EMC	0.65	5.73	2.19	4.7	0.56	0.52	3.61	1.27	4.86	8.04	1.55	3.32
9	3-EEC	8.7	7.62	2.22	1.06	0.42	0.34	5.69	4.88	2.1	4.18	2.44	2.22
10	4-EEC	4.38	5.72	7.91	5.68	2.01	2.32	2.37	12.25	0.66	6.64	0.9	1.52
11	3,4-DMEC	4.36	3.85	2.2	2.46	1.89	2.93	9.61	7.83	7.35	8.05	2.07	1.64
12	2,3-MDMC	9.98	8.22	6.17	8.34	1.97	3.51	7.25	10.31	9.45	6.68	0.94	0.54
13	Butylone	1.41	10.98	2.05	5.97	1.62	1.98	2.93	3.78	8.95	8.38	0.83	0.24
14	Pentylone	5.8	10.43	1.07	1.47	1.68	0.22	9.54	0.05	0.14	3.25	2.52	3.14
